# Dataset of genomic sequences and annotation of *Bacillus cereus* Spbamp1 isolated from metal-impacted soil in Querétaro, México

**DOI:** 10.1016/j.dib.2026.112853

**Published:** 2026-05-14

**Authors:** Mario Eduardo Clemente Albores, Mayra Paola Mena Navarro, Karla Isabel Lira De León, David Gustavo García Gutiérrez, Miguel Ángel Ramos López, Carlos Eduardo Zavala-Gómez, José Alberto Rodríguez Morales, Erika Álvarez Hidalgo, Juan Campos Guillén

**Affiliations:** a Facultad de Química, Universidad Autónoma de Querétaro, Cerro de las Campanas S/N, Querétaro 76010, México; b Centro de Investigación en Materiales Semiconductores, Sustentabilidad y Energía Renovable (CIMSSER), Facultad de Química, Universidad Autónoma de Querétaro, Cerro de las Campanas S/N, Querétaro 76010, México; c Facultad de Ingeniería, Universidad Autónoma de Querétaro, Cerro de las Campanas S/N, Querétaro 76010, México

**Keywords:** Bacillus cereus, Whole genome sequencing, Antimicrobial resistance genes, Virulence genes, Heavy metal resistance genes

## Abstract

*Bacillus cereus* is a Gram-positive bacterium with the ability to form spores and is found in different environments such as food and soil. We report the complete genome sequence of *B. cereus* Spbamp1 strain, isolated from soil contaminated with leachates in Querétaro, Mexico. The complete genome was sequenced using the Illumina Novaseq platform and 51 contigs were assembled using the BV-BRC platform, with a total of 5547,101 base pairs, GC content of 34.91%, and 5712 predicted protein-coding sequences. The Spbamp1 strain was shown to be closely related to *B. cereus* ATCC 14,579 with ANI values of 96.08% and dDDH values of 79.06%, confirming its taxonomic analysis. This whole-genome dataset represents a valuable tool for investigating the genetic determinants of virulence and antimicrobial resistance in this environmental strain of *B. cereus*. In addition, it helps to understand the possible mechanisms of co-selection between heavy metal tolerance and antibiotic resistance in contaminated environments.

Specifications Table

**Table:** 

Subject	Biological sciences
Specific subject area	Microbiology, Genomics, Bioinformatics
Data format	Raw, Filtered and analyzed
Type of data	Complete genome sequence in FASTA format Figure(s) Table(s)
Data collection	*Bacillus cereus* Spbamp1 was isolated from soil contaminated by leachate with high concentrations of heavy metals in Querétaro, Mexico. Genomic DNA extraction and purification was performed using the ZymoBIOMICS™ DNA Miniprep Kit. Bioinformatic tools: Platform BV-BRC, Trim Galore v0.6.5dev, Fastq-Pair, SPAdes, RASTtk, QualiMap (BamQC module), Mash/MinHash, RaxML, JspeciesWS, Type (Strain) Genome Server (TYGS), plasmidSPAdes, PlasmidScope.
Data source location	Institution: Universidad Autónoma de Querétaro City/Town/Region: Querétaro, Qro. Country: México GPS coordinates: 20°60′N 100°39′W
Data accessibility	The assembly data is deposited in a public repository, and the data analyzed are presented in this report. Repository name: NCBI. Data identification number: JBVAYO000000000 Bio Project: PRJNA1424671 Bio Sample: SAMN55391293 Direct URL to data: https://www.ncbi.nlm.nih.gov/nuccore/JBVAYO000000000 https://www.ncbi.nlm.nih.gov/bioproject/PRJNA1424671 https://www.ncbi.nlm.nih.gov/biosample/ SAMN55391293

## 1 Value of the Data

• The whole-genome sequencing of *Bacillus cereus* Spbamp1 provides high-resolution genetic information that enhances taxonomic accuracy and enables robust phylogenomic comparisons with related strains isolated from heavy metal-contaminated soils.
• The dataset contributes to a deeper understanding of the genomic architecture of this species associated with heavy metal-contaminated soil, including genome size, GC content, coding potential, and gene repertoire.
• This genomic data allows the identification and comparative analysis of genes associated with virulence, stress response, metal resistance genes, and antimicrobial resistance, supporting risk assessment from both environmental and food safety perspectives.
• This genome data set serves as a robust resource for comparative genomic analyses, epidemiological tracking, and functional investigations aimed at understanding metabolic capabilities and environmental adaptability.

## 2 Background

*Bacillus cereus* is a Gram-positive, endospore-forming bacterium [1] commonly found in natural environments such as soil. It is a member of the *Bacillus cereus sensu lato* group, characterized by high genomic similarity among species with diverse ecological and pathogenic profiles [2]. The ability to form spores allows it to survive in adverse environmental conditions, such as soil contaminated with heavy metals and other xenobiotics [3]. *Bacillus cereus* is an opportunistic pathogen and a significant cause of foodborne illness, causing emetic and diarrheal syndromes. In addition, it can cause serious invasive infections, such as bacteremia, meningitis, and endophthalmitis, particularly in immuno-compromised patients. [4]. The virulence of *Bacillus cereus* is attributed to the production of different factors that favor interaction with the host, allowing it to persist in adverse environments. Furthermore, its resistance to beta-lactams could be a challenge for effective clinical treatment [5]. From an environmental point of view, the high frequency of *B. cereus* in contaminated soils indicates a remarkable ability to adapt to chemically stressed environments, such as exposure to heavy metals [6]. This bacterium can survive in these environments due to physiological mechanisms that reduce metal toxicity, such as cellular tolerance, metal immobilization, and prolonged survival through spore formation [7]. These adaptations not only favor their permanence in contaminated ecosystems but may also contribute to their dissemination and the selection of strains with higher survival capacity [8,9]. In this context, the investigation of environmental strains of *B. cereus* is crucial to understanding the relationship between ecological adaptability, resistance to environmental stress factors, and their possible impact on public health.

## 3 Data Description

**Fig. 1:**
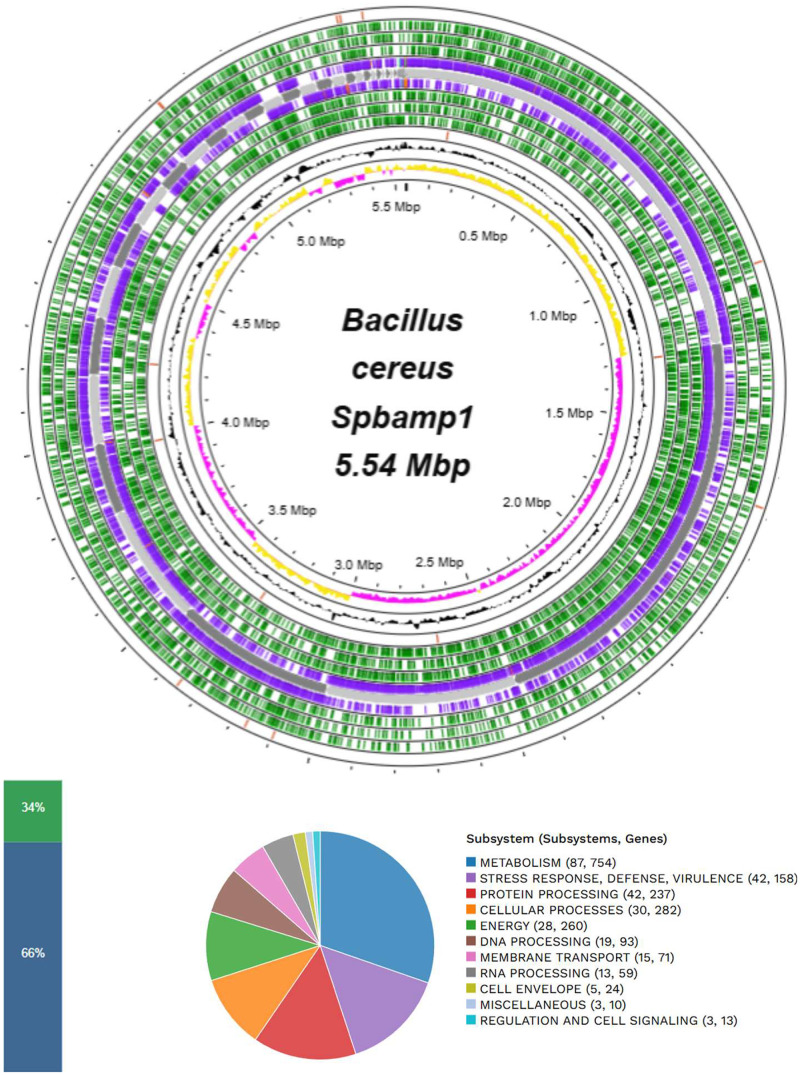
Subsystem information and circular genomic map of *B. cereus* Spbamp1. From the outside to the center are the contigs assembled, the open reading frame (ORF), the CDS in the forward strand, the CDS in the reverse strand, the RNA genes, the CDS with a similarity to known antibiotic resistance genes, the CDS with a similar virulence factor, the GC content, and the GC bias. In the coverage of the subsystems, 66% represents a total of 3834 genes, and 34% is represented by those not indicated in the subsystem average, with a total of 1961 genes.

Table 1 shows the genomic features of *Bacillus cereus* Spbamp1 that we got from the BV-BRC platform. The assembled genome was 5547,101 bp. The assembly generated 51 contigs, with an N50 value of 540,449 bp and an overall G + C content of 34.91%. Genome annotation predicted 5712 protein-coding sequences (CDS), as well as 4 ribosomal RNA (rRNA) genes and 79 transfer RNA (tRNA) genes. Functional analysis also identified 1540 hypothetical proteins. In addition, according with the database source, the genes were related to predicted drug targets, virulence-associated genes, transport-related genes, and antibiotic resistance-linked genes. The distribution of functional subsystems related to the fundamental biological and metabolic activities necessary for bacterial survival is presented in Fig. 1. In addition, plasmid-associated sequences were detected in three contigs of the *B. cereus* Spbamp1 genome (Table 2), which showed significant alignments against plasmids from related members of *Bacillus cereus* group (Table 3). Contig 1 was 139,440 bp in length with a G + C content of 31.91% and included 117 CDSs and 2 tRNA genes. Contig 2 was 54,700 bp long with a G + C content of 35.96% and contained 89 CDS. Contig 3 was 12,086 bp long with a G + C content of 34.29% and encoded 18 CDS.

**Table 1:** Genomic description of *Bacillus cereus* Spbamp1.

Characteristics	Source	Total
**Genome Length**	PATRIC	5547,101 bp
**Number of contigs**	PATRIC	51
**Number of total genes**	PATRIC	5681
**Number of CDS´s**	PATRIC	5384
**Number of rRNA genes**	PATRIC	4
**Number of tRNA genes**	PATRIC	78
**Number of ncRNA genes**	PATRIC	5
**G+C**	PATRIC	34.91%
**N50 contig size**	PATRIC	540,449 bp
**Virulence factor**	VFDB	9
**Virulence factor**	VICTORS	13
**Antibiotic resistance genes**	NDARO	8
**Antibiotic resistance genes**	CARD	9
**Antibiotic resistance genes**	PATRIC	58
**Transporter genes**	TCDB	49
**Drug target genes**	DrugBank	27
**Drug target genes**	TTD	1

**Table 2:** Putative plasmid contigs identified in *Bacillus cereus Spbamp1*.

Genome features	Plasmid 1	Plasmid 2	Plasmid 3
**Genome size**	139,440 bp	54,700 bp	12,086 bp
**GC contents**	32.91%	35.96%	34.29%
**Total genes**	119	89	18
**CDSs**	117	89	18
**tRNA**	2	0	0
**Specialty genes**	0	0	0

**Table 3:** Genomic sequences producing significant alignments against plasmid sequences of *Bacillus cereus* Spbamp1.

Plasmid	Sequence with significant alignment	Query cover	Percent identity	Length	Sequence ID
**1**	*Bacillus cereus* G9842 plasmid pG9842_140	97%	96.41%	193,440 bp	CP001188.1
**2**	*Bacillus cereus* strain SEM-15 plasmid pb3	42%	92.44%	54,700 bp	CP095380.1
**3**	*Bacillus mycoides* strain PAMC 29,501 plasmid unnamed3	25%	94.51%	12,086 bp	CP136384.1

Genome analysis identified eight antibiotic resistance mechanisms in the studied bacterial isolate: (1) efflux pumps (*BcrA, BcrB, YkkCD*); (2) antibiotic target protection proteins (*BcrC, Lsa(B)*); (3) cell wall structure modification (*VanXY*); (4) antibiotic inactivation enzymes (*BclI, CatA15/A16, FosB*); (5) resistance via gene absence (*gidB*); (6) regulatory systems (*LiaF, LiaR, LiaS, VanA/I/Pt-type, VanF/M-type, VanR*); (7) cell wall charge modification (*GdpD, MprF, PgsA*); and (8) antibiotic target modification in susceptible species (*gyrA, gyrB, rpoB, rpoC, MurA, kasA, fabI, folA, folP, Dfr, Alr, Ddl, inhA, dxr, EF-G, EF-Tu, Iso-tRNA, rho, S10p, S12p*). Heavy metal resistance genes were also identified: arsenic (*arsR,arsC,acr3*), cadmium (*cadA,cadC*), copper (*copC,copD,copZ*) and cobalt-zinc-cadmium (*czcABC, czcD*).

**Fig. 2:**
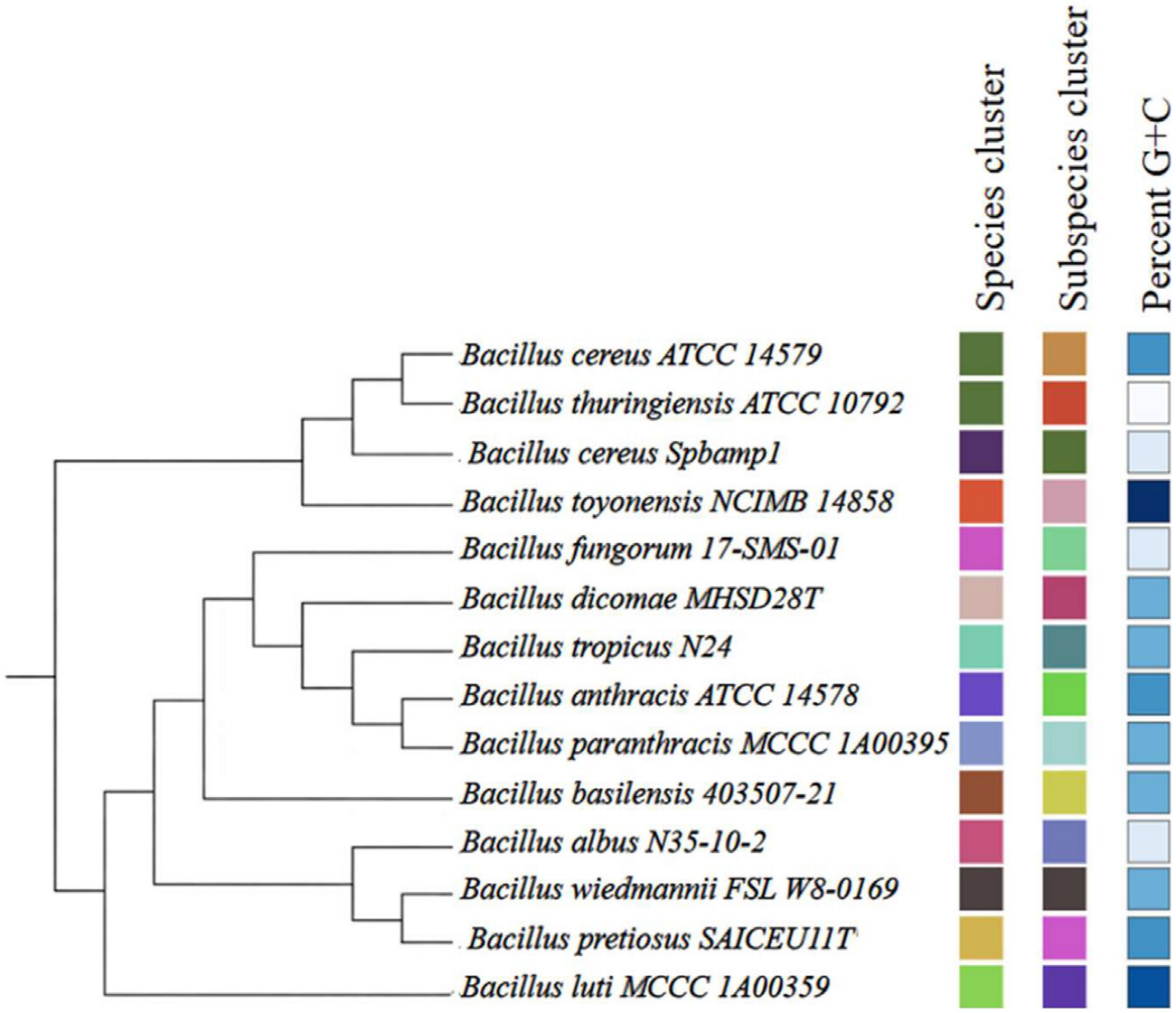
Results from TYGS for the dataset. The phylogenomic tree was inferred using FastME 2.1.456 based on GBDP distances calculated from genome sequences. Branch lengths are scaled according to the GBDP distance formula d5, and numbers above the branches represent GBDP pseudo-bootstrap support values from 100 replicates.

**Fig. 3:**
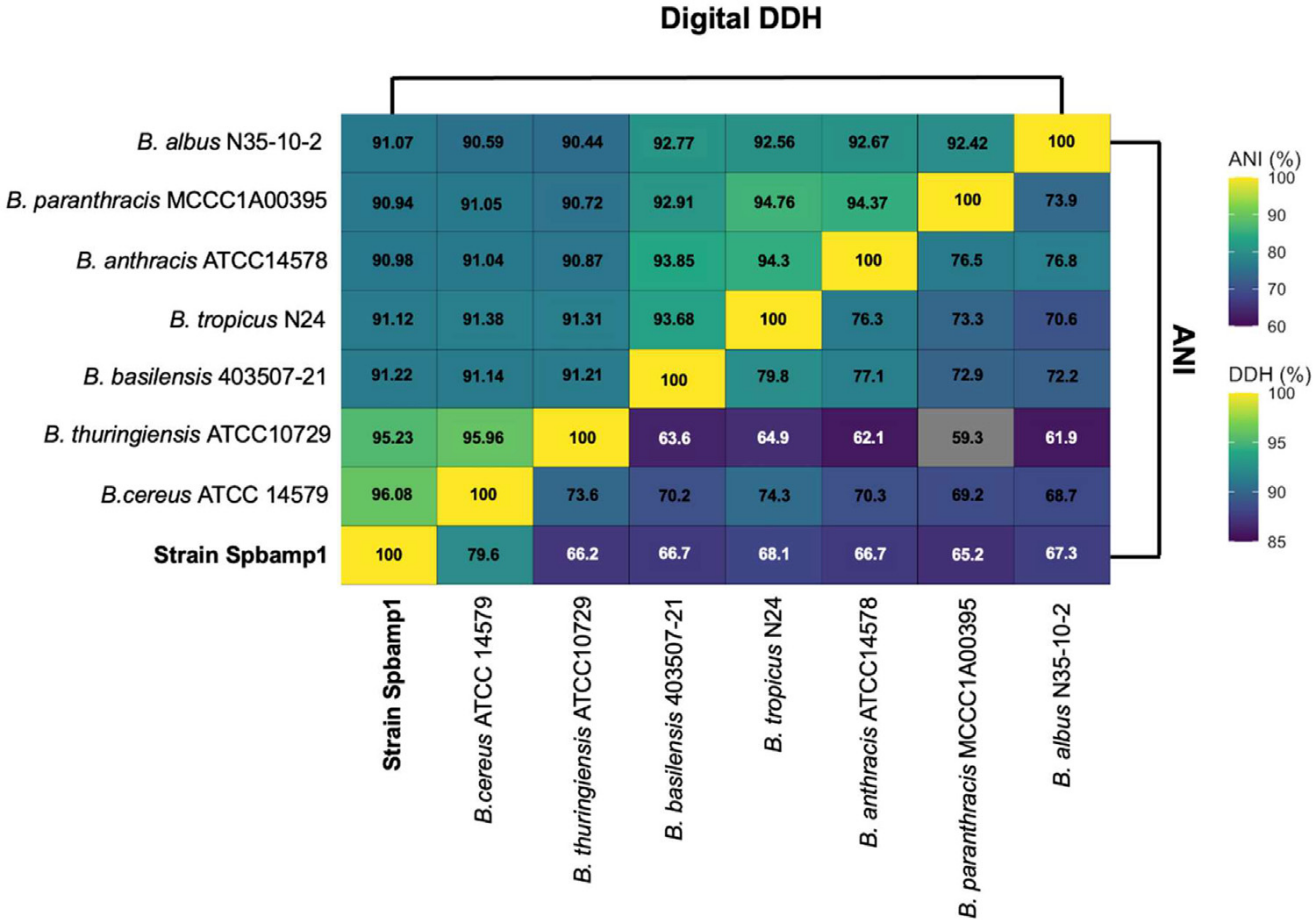
Heat maps representing digital DNA-DNA hybridization (dDDH) values and average nucleotide identity (ANI) for strain Spbamp1 and closely related *Bacillus* strains.

Table 4 shows the genes related to virulence according to the source of bacterial virulence factors (VDFB). Fig. 2 presents a phylogenomic tree based on complete genomes of the *Bacillus cereus sensu lato* complex, accompanied by a matrix of comparative genomic annotations, where the Spbamp1 strain is closely related to *Bacillus cereus* ATCC 14,579 and *Bacillus thuringiensis* ATCC 10,792. Comparative pairwise analysis of the Spbamp1 genome revealed an ANI value of 96.08% with *B. cereus* ATCC 10,792 (95% cutoff). dDDH analysis indicated a value of 79.6% for the same comparison (70% cutoff). These results support its classification as Bacillus cereus Spbamp1 in Fig. 3.

**Table 4:** Predicted virulence-related genes in *Bacillus cereus* identified through VFDB analysis.

Gen	Product	Mechanism of action
***inhA***	*Immune inhibitor A, metalloprotease*	Metalloprotease degrades antibacterial peptides and proteins of the host; facilitates evasion of the immune system and bacterial dissemination.
***nheA***	*Non-hemolytic enterotoxin A*	It binds to the cell membrane after sequential interaction with NheB and NheC, participating in the formation of pores that alter cell permeability.
***nheB***	*Non-hemolytic enterotoxin lytic component L1*	First component that binds to the host cell membrane; acts as an initial receptor and allows the recruitment of NheC and subsequently NheA for pore formation.
***nheC***	*Enterotoxin C*	Component that stabilizes the Nhe complex in the membrane and enhances the insertion of NheA, contributing to the functional formation of the pore.
***BAS3109***	*Thiol-activated cytolysin*	Thiol-dependent cytolysin dependent on membrane cholesterol; forms large pores in eukaryotic cells, causing cell lysis and tissue damage.
***cytK***	*Cytotoxin K*	Pore-forming toxin that induces enterocyte lysis; associated with severe diarrhea and intestinal necrosis.
***hblA***	*Hemolysin BL component B*	Initial binding component of the Hbl complex; it attaches to the host cell membrane and allows the assembly of the toxigenic complex.
***hblC***	*Hemolysin BL lytic component L2*	A lytic component that, together with HblD, participates in the formation of pores in the cell membrane, causing lysis.
***hblD***	*Hemolysin BL lytic component L1*	Essential component of the Hbl complex that completes the assembly of the transmembrane pore and causes osmotic destabilization and cell lysis.

Antimicrobial resistance of the strain was assessed on Mueller–Hinton agar, showing resistance to β-lactams (ampicillin, penicillin, dicloxacillin, carbenicillin, cephalothin, cefotaxime) and sulfonamides (sulfamethoxazole/trimethoprim). The bacterial strain was sensitive to fluoroquinolones (ciprofloxacin, norfloxacin), aminoglycosides (amikacin, gentamicin, netilmicin), macrolides (erythromycin), lincosamides (clindamycin), glycopeptides (vancomycin), phenicols (chloramphenicol), nitrofurans (nitrofurantoin) and tetracyclines (tetracycline).

## 4 Experimental Design, Materials and Methods

### 4.1 Sample collection and microbial isolation

The soil samples were obtained from a landfill located in Querétaro, México (20° 24′ 18″ N, 100° 01′ 12″ W). A quadrat-based random sampling method was used to collect soil samples from a contaminated area of 26,148 m^2^, previously delimited using geographic coordinates and GIS tools. The area was divided into equal-sized quadrats using a grid system, and three quadrants were randomly selected to ensure unbiased spatial representation. From each selected quadrat, 1 kg of soil samples were collected at a depth of 0–30 cm, after removing surface debris. Multiple subsamples (3–5) were taken within each quadrant and homogenized to form one composite sample per quadrant. The samples were stored in sterile containers and transported for further processing. The concentration of heavy metals in this area was previously described and quantified [10]. As first approach to isolate ampicillin-resistant bacteria, soil samples (1 g) were suspended in 10 mL of sterile water and subjected to serial dilutions ranging from 10⁻¹ to 10⁻³. Aliquots of these dilutions were spread on Mueller–Hinton agar plates containing ampicillin (100 µg/mL) and incubated at 37 °C for 24 h. After incubation, colony-forming units per gram of soil (CFU/g) were quantified (average of 4 × 10^2^ CFU/g), and bacteria colony were characterized based on their morphologies features and subsequently isolated to obtain pure culture. Each isolate was tested for antibiotic susceptibility (described below). Based on these results, similar phenotypic isolates showed resistance to β-lactams tested, one of them identified as spbamp1, was selected for genomic sequencing. The bacterial isolated spbamp1 was preserved in sterile glycerol at 20% and stored at −80 °C for long-term maintenance in the laboratory strain collection.

### 4.2 Genome sequencing, assembly and annotation

The Shotgun Genomic Sequencing Service (Zymo Research, Irvine, CA) was used to obtain the genomic DNA from spbamp1 strain. Then, sequencing libraries were prepared with Illumina® DNA Library Prep Kit (Illumina, San Diego, CA) with up to 500 ng DNA input following the manufacturer’s protocol using unique dual-index 10 bp barcodes with Nextera® adapters (Illumina, San Diego, CA) and the final library was sequenced on the platform Illumina® NovaSeq (Illumina, San Diego, CA). The raw paired-end sequences (total reads processed: 79,828,892) were trimmed using Trim Galore v0.6.5dev, Cutadapt version 4.9, applying a maximum error rate of 0.1, a minimum adapter overlap of 1 bp, a Quality Phred score cutoff of 20 and a minimum length of 20 bp per read before removing incomplete pairs [11]. A sequencing quality score of Q30, reads with a quality score of 39 (Phred score) and read lengths ranging from 20 to 151 bp were obtained in FastQC (version 0.12.1). The filtered sequences were subsequently reconciled with Fastq-Pair and assembled de novo into contigs with SPAdes genome assembler v4.0.0. The filtering contigs on length and coverage were: min. contig coverage threshold: 5.0; min. contig length threshold: 300 and average depth (short reads): 351.65. Genome annotation was performed using PATRIC alongside RASTtk [11,12]. The genome quality metrics obtained were: Contig L50: 6, Contig N50: 540,449 bp, Coarse Consistency (99.9%), Fine Consistency (99.1%), and CheckM Completeness (100%). Alignment quality and genome coverage were evaluated using QualiMap (BamQC module) in Galaxy platform [13] from a sorted and indexed BAM file mapped to a 5547,101 bp reference genome. A total of 56,807,166 reads were analyzed, all of which mapped successfully (100%), showing high alignment confidence (mean mapping quality=37.43). Coverage metrics indicated 8.51 Gb of mapped bases, with a high duplication rate (83.02%), reducing the effective depth to approximately 250x and a genome fraction coverage of 100%, demonstrating complete representation of the reference genome. Insert size analysis showed a mean of 276.7 bp (median 232 bp) with high variability and a large number of overlapping read pairs, indicating redundancy in the data set; nevertheless, the effective coverage and full genome fraction support the reliability of the sequencing data for downstream genomic analyses. The genome was visualized as a circular map with Proksee [14], while species-level relatedness was evaluated through average nucleotide identity (ANI) using JspeciesWS [15,16] and digital DNA–DNA hybridization (dDDH) [17] and Phylogenomic tree was constructed using the Type (Strain) Genome Server (TYGS) [18]. Plasmids were assembled from the sequenced reads using the plasmidSPAdes algorithm [19], followed by annotation with the PlasmidScope platform [20]. Finally, a plasmid database was queried to identify similar plasmids [21]. All software analyses were performed using default parameters. The complete whole-genome shotgun assembly is available in NCBI under accession JBVAYO000000000, linked to BioProject PRJNA1424671 and BioSample SAMN55391293.

### 4.3 Antibiotic sensitivity test

Antimicrobial susceptibility of *Bacillus cereus strain Spbamp1* was evaluated using the disk diffusion method. The antibiotics tested (disk content) were: β-lactams (ampicillin 10 µg, penicillin 10 µg, dicloxacillin 1 µg, carbenicillin 100 µg, cephalothin 30 µg, cefotaxime 30 µg), sulfonamides (sulfamethoxazole/trimethoprim 25 µg), fluoroquinolones (ciprofloxacin 5 µg, norfloxacin 10 µg), aminoglycosides (amikacin 30 µg, gentamicin 10 µg, netilmicin 30 µg), macrolides (erythromycin 15 µg), lincosamides (clindamycin 30 µg), glycopeptides (vancomycin 30 µg), phenicols (chloramphenicol 30 µg), nitrofurans (nitrofurantoin 300 µg), and tetracyclines (tetracycline 30 µg).

## Limitations

Not applicable.

## Ethics Statement

This work does not involve human subjects or animal subjects.

## CREDIT Author Statement

**Mario Eduardo Clemente Albores:** Writing-original draft, Methodology; **Mayra Paola Mena Navarro:** Writing-original draft, Methodology; **Karla Isabel Lira De León:** Writing- original draft, Resources, Methodology; **David Gustavo García Gutiérrez:** Writing-review & editing; **Miguel Ángel Ramos López:** Writing-review & editing; **Carlos Eduardo Zavala-Gómez:** Resources, Methodology, **José Alberto Rodríguez Morales:** Writing-review & editing; **Erika Álvarez Hidalgo:** Conceptualization, Methodology; **Juan Campos Guillén:** Validation, Supervision, Resources, Writing-review & editing, Supervision.

## Data Availability

NCBIBacillus cereus strain spbamp1, whole genome shotgun sequencing project (Original data).
